# At the Dawn of Applied DNA Nanotechnology

**DOI:** 10.3390/molecules25030639

**Published:** 2020-02-03

**Authors:** Veikko Linko

**Affiliations:** 1 Biohybrid Materials, Department of Bioproducts and Biosystems, Aalto University, P.O. Box 16100, 00076 Aalto, Finland; veikko.linko@aalto.fi; Tel.: +358-45-673-9997; 2 HYBER Center of Excellence, Department of Applied Physics, Aalto University, 00076 Aalto, Finland

Deoxyribonucleic acid (DNA) serves not only as a genetic information carrier but also as an excellent material for programmable nanoscale assembly [1,2]. The research field dubbed DNA nanotechnology has evolved significantly from simple structural motifs [3] to the current enabled state with ever-expanding shape space [4]. There is an extensive choice of methods for designing custom, user-defined, and accurate DNA nanoarchitectures for a number of fascinating functions [5], with DNA origami [6,7,8] being the most commonly employed technique. The various experimental procedures have emerged hand in hand with design software development [9], and, currently, the most prominent classes of DNA structures are based on lattices [10], molecular canvases [11], or wireframe constructions [12,13]. Some design methods are fully automated [14], and, arguably, this kind of advancement would encourage researchers to adopt easily accessible state-of-the-art approaches for their own laboratory toolbox. Importantly, it has also been shown that the extreme subnanometer-level precision of these objects [15] may be connected to larger length scales without a trade-off between the accuracy and dimensions of the system [16,17,18]. Apart from that, the upgraded and scaled-up production of DNA structures based on cost-effective biotechnological mass production has, remarkably, knocked down the price of synthesis [19]. Together with static and more complex objects, dynamic and functional DNA structures are increasingly coming into view [20,21], thus opening a route for sophisticated device fabrication for implementations in, e.g., biomedicine [22,23,24], super-resolution imaging [25,26], optical and plasmonic devices [27,28,29,30,31], and robotics [32,33,34]. In other words, we are currently at the dawn of a new era, looking forward to the bright future of applied DNA nanotechnology.

This Special Issue of *Molecules* entitled “*Emerging Trend in DNA Nanotechnology*” covers some of the key topics and challenges on our way toward real-life applications of DNA nanostructures. Dr. Katherine E. Dunn (University of Edinburgh, UK) summarizes, in her article, the existing and upcoming trends using data from publications and patent applications as indicators of expanding activities in the field [35]. This illustrative and intriguing article emphasizes the great promise of DNA nanotechnology and introduces examples of start-ups and spin-offs, while the real breakthrough in commercialization is still to be achieved.

During the past decade, most publications in the field have presented new types of DNA objects along with novel techniques and practices for effortless fabrication. It could be argued that it was a decade of learning how to build structures, and it is foreseen that the next one will be an era of *doing something* with them, including the development of new functionalities. One substantial subfield toward this research direction is to combine DNA nanostructures with active proteins [36,37]. In an extensive review of this issue, Prof. Barbara Saccà and her co-workers (University Duisburg-Essen, Germany) describe and list techniques to modulate and manipulate the properties of enzymes with the help of DNA structures [38]. I believe this article will be extremely useful for many researchers working on hybrid DNA-protein structures.

In the experimental research section of this issue, Prof. Shin-ichiro M. Nomura (Tohoku University, Japan) and colleagues report a refolding process of DNA origami in water-in-oil droplets, thus providing interesting insights into the self-assembly processes taking place in micron-sized compartments [39]. In another fundamental study, Prof. Ilko Bald’s group (University of Potsdam, Germany) demonstrates how amorphous carbon is formed on discrete DNA origami-templated silver and gold nanoparticle aggregates when exposed to laser light [40]. These results may help researchers to find ways to prevent undesired amorphous carbon formation on widely employed DNA-based surface-enhanced Raman scattering (SERS) substrates.

Finally, a research led by Dr. Adrian Keller (Paderborn University, Germany) shows that the age of short DNA strands forming DNA origami may affect the quality of origami folding and their stability, especially in harsh experimental conditions [41]. The work is a logical continuation of the previous studies in this topical field exploring the (often superstructure-dependent) stability of DNA nanostructures in various challenging conditions [42,43] involving low-magnesium buffers [44] and other biologically relevant media, e.g., an endonuclease-rich environment [45] and in vivo experimentation [46].

As an Academic Guest Editor of this Special Issue, I would like to express my thanks to all the authors for their valuable contributions. I would also like to thank the editorial staff of *Molecules* and the reviewers of the manuscripts for their efforts and precious time. I hope you will find this collection of articles beneficial and inspiring.
